# Genome of a Novel Bacterium “*Candidatus* Jettenia ecosi” Reconstructed From the Metagenome of an Anammox Bioreactor

**DOI:** 10.3389/fmicb.2019.02442

**Published:** 2019-10-29

**Authors:** Andrey V. Mardanov, Alexey V. Beletsky, Nikolai V. Ravin, Ekaterina A. Botchkova, Yuriy V. Litti, Alla N. Nozhevnikova

**Affiliations:** ^1^Institute of Bioengineering, Research Center of Biotechnology, Russian Academy of Sciences, Moscow, Russia; ^2^Winogradsky Institute of Microbiology, Research Center of Biotechnology, Russian Academy of Sciences, Moscow, Russia

**Keywords:** anammox, metagenome, bioreactor, “*Candidatus* Jettenia ecosi”, FISH

## Abstract

The microbial community of a laboratory-scale bioreactor based on the anammox process was investigated by using metagenomic approaches and fluorescent *in situ* hybridization (FISH). The bioreactor was initially inoculated with activated sludge from the denitrifying bioreactor of a municipal wastewater treatment station. By constantly increasing the ammonium and nitrite load, a microbial community containing the novel species of anammox bacteria “*Candidatus* Jettenia ecosi” developed in the bioreactor after 5 years when the maximal daily nitrogen removal rate reached 8.5 g/L. Sequencing of the metagenome of anammox granules and the binning of the contigs obtained, allowed a high quality draft genome of the dominant anammox bacterium, “*Candidatus* Jettenia ecosi” to be assembled. Annotation of the 3.9 Mbp long genome revealed 3970 putative protein-coding genes, 45 tRNA genes, and genes for 16S/23S rRNAs. Analysis of the genome of “*Candidatus* Jettenia ecosi” revealed genes involved in anammox metabolism, including nitrite and ammonium transporters, copper-containing nitrite reductase, a nitrate reductase complex, hydrazine synthase, and hydrazine dehydrogenase. Autotrophic carbon fixation could be accomplished through the Wood Ljungdahl pathway. The composition of the community was investigated through a search of 16S rRNA sequences in the metagenome and FISH analysis of the anammox granules. The presence of the members of Ignavibacteriae, Betaproteobacteria, Chloroflexi and other microbial lineages reflected the complexity of the microbial processes in the studied bioreactor performed by anammox Planctomycetes, fermentative bacteria, and denitrifiers.

## Introduction

Anammox bacteria are unique autotrophic members of Planctomycetes capable of anaerobic ammonia oxidation with nitrite (Strous et al., 1999). Since their discovery, anammox bacteria are considered to be biotechnologically valuable microorganisms. They are successfully applied in various systems for effective nitrogen removal, including wastewater and leachate treatment (Kartal et al., 2010; Cao et al., 2017; Ye et al., 2019). Anammox bacteria produce dinitrogen gas via anammox process: anaerobic ammonia oxidation with nitrite. The anammox process has three stages: nitrite reduction to NO catalyzed by nitrite reductase; synthesis of hydrazine (N_2_H_4_) from NO and ammonia carried out by hydrazine synthase and oxidation of hydrazine to dinitrogen, hypothetically by a variant of hydroxylamine oxidoreductase (Kartal and Keltjens, 2016). To date, five candidate genera of anammox bacteria have been described: “*Candidatus* Brocadia, Kuenenia, Scalindua, Jettenia, and Anammoxoglobus.” First member of the genus “*Candidatus* Jettenia,” “*Ca.* Jettenia asiatica,” was described in 2008 (Quan et al., 2008). Members of the genus “*Ca*. Jettenia” are typical inhabitants of bioreactors (Cho et al., 2017; Yang et al., 2018; Chini et al., 2019), they also were found in natural environments such as soils, groundwater and freshwater sediment (Lisa et al., 2014; Sonthiphand et al., 2014; Zhao S. et al., 2018). The first member of the genus “*Candidatus* Jettenia,” “*Ca.* Jettenia asiatica,” was detected in a granular sludge anammox reactor (Quan et al., 2008). According to the analysis of metagenome of this bioreactor, “*Ca.* Jettenia asiatica” was predicted to have a flexible anammox metabolism, close to that in other anammox bacteria, and possesses an ability to fix carbon via the Wood Ljungdahl pathway (Hu et al., 2012). Among the members of the genus “*Ca.* Jettenia,” near-complete genome sequence was reported only for “*Ca.* Jettenia caeni” KSU-1, obtained in an enrichment culture (Ali et al., 2015), but this genome has not been analyzed in details.

A novel species of the genus, “*Ca*. Jettenia ecosi,” was described in 2018 as a key member of microbial community of laboratory-scale upflow bioreactor fed with mineral medium with elevated nitrogen load (Botchkova et al., 2018). “*Ca*. Jettenia ecosi” is active in a wide range of substrate concentrations (0.02–5.6 g N/L), it can tolerate pH levels from 7.2 to 8.8 and microaerophilic conditions (presence of 3% oxygen in the gas phase). In the current article we report the results of the metagenomic studies of bioreactor community, metagenome sequencing and analysis, combined with fluorescent *in situ* hybridization (FISH) analysis.

## Materials and Methods

### Enrichment of Anammox Biomass in a Lab-Scale Anammox Bioreactor

To obtain an active microbial community enriched with anammox bacteria, flow cultivation in a vertical lab-scale upflow bioreactor was used. The bioreactor was initially inoculated with activated sludge from a denitrificator of a wastewater treatment plant “BCH-ECOS” constructed by the “ZAO ECOS” company in the Sochi region of Russia. Start-up and construction of the bioreactor has been described previously (Nozhevnikova et al., 2012). The bioreactor was fed with a mineral medium containing NH_4_Cl and NaNO_2_ in the molar ratio of 1:1.32 as substrates for the anammox process. Concentrations of substrates were gradually increased as the biomass became adapted to it. By the 5th year of cultivation, i.e., the time when the metagenomic studies were carried out, the nitrogen load had reached 8.5 g N⋅L^–1^⋅day^–1^. Working conditions of the bioreactor are summarized inTable 1.

**TABLE 1:** Working conditions of a bioreactor for biomass enrichment by the start of metagenomic studies.

**Indicator**	**Value**
N-NH4+, mg/L	400 ± 15
N-NO2−, mg/L	400 ± 20
Temperature, °Ñ	30
pH of synthetic media	7.6–7.8
Oxygen concentration in synthetic media, mg/L	2.01–2.88
Working volume of the bioreactor, L	0.8
Flow rate, L/day	6–8

The bioreactor was equipped with polymer brush-shaped carriers for biomass immobilization.

### Chemical Analyses

Concentrations of nitrite and ammonium were measured colorimetrically using a Hach Lange DR 5000 (“Hach,” Germany) spectrophotometer according to standard methods proposed by the manufacturer: NH_4_^+^ – with Nessler reagent, NO_2_^–^ – with Griess reagent and using the ferrous sulfate reduction method. Oxygen concentration was measured with a Seven2Go Pro portable oxygen meter, with an InLab OptiOX sensor (“Mettler Toledo,” Switzerland). pH was measured using a HANNA pH-211 (Germany) laboratory pH-meter.

### Fluorescent *in situ* Hybridization

Fixation and pre-treatment of samples were conducted as described previously (Botchkova et al., 2014). In brief, samples were fixed in a 4% paraformaldehyde/phosphate-buffered saline solution and applied onto slides coated with a 0.1% gelatine solution containing 0.01% KCr(SO_4_)_2__._ Post fixation in 50, 80, and 96% ethanol was performed (3 min each). Hybridization was carried out at a temperature of 46°Ñ according to the standard scheme (Amann et al., 1990). All of the Cy3-labeled oligonucleotide probes used in this study, together with their specificity and hybridizing conditions, are listed in table (Supplementary Table S1). Cy-3-labeled probes were provided by “Syntol” (Russia).

### Microscopy

Phase contrast and epifluorescent microscopy were carried out using a Zeiss Lab.A1 (“CarlZeiss,” Germany) microscope, equipped with an AxioCamHR digital camera, with a Zeiss 20 filter for Cy-3-labeled probes for FISH.

### Metagenome Sequencing and Assembly, Contig Binning, and Analysis of the Composite Genome of the Anammox Bacterium

The red-colored anammox granules were collected from the bioreactor, washed in water and used for DNA extraction (Supplementary Figure S1). Metagenomic DNA was isolated from anammox granules using the PowerSoil DNA isolation kit (Mo Bio, Inc., Laboratories, Carlsbad, CA, United States) and sequenced with a Roche Genome Sequencer (GS FLX), using the Titanium XL + protocol for a shotgun genome library. About 682.6 Mb of sequences with an average read length of 411 nt were generated and *de novo* assembled into contigs using the Newbler Assembler version 2.9 (454 Life Sciences, Branford, CT, United States) with default settings.

Contigs longer than 1000 bp were binned into clusters representing the metagenome-assembled genomes (MAG) of the community members using the program CONCOCT v. 0.4.1 (Alneberg et al., 2014). For “*Ca.* Jettenia ecosi” MAG the correctness of binning was manually curated using the Newbler assembly graph which shows the connections of contigs to each other and coverage information. We manually checked contigs that contained no genes with high similarity (more than 90% amino acid sequence identity) to “*Ca.* Jettenia caeni.” If such contig had graph edges only with other contigs from “*Ca.* Jettenia ecosi” bin at least at one end we kept it. If such questionable contig had edges with contigs from other genome bins we removed it. If the contig has the similar sequencing coverage like other contigs from “*Ca.* Jettenia ecosi” bin and no graph edges to other contigs we kept it. Finally, five contigs were excluded from the CONCOCT-generated “*Ca.* Jettenia ecosi” MAG initially comprised 228 contigs.

The completeness and contamination of the recovered MAGs were estimated using CheckM v. 1.05 (Parks et al., 2015) with lineage-specific marker genes. The assembled MAGs were taxonomically assigned using GTDB-Tk version 0.1.3 tool and Genome Taxanomy database (GTDB) (Parks et al., 2018).

The 16S rRNA genes were found in binned contigs by CheckM. For MAG assigned to the Candidatus Jettenia sp. J2 gene search and annotation were performed using the RAST server 2.0 (Brettin et al., 2015), followed by manual correction by searching the National Center for Biotechnology Information (NCBI) databases. Signal peptides were predicted using Signal P v.4.1 for Gram-negative bacteria^1^ and PRED-TAT^2^. The transmembrane helices were predicted with TMHMM Server v. 2.0^3^.

The values of DNA-DNA hybridization *in silico* were calculated using GGDC 2 (Meier-Kolthoff et al., 2013), available at http://ggdc.dsmz.de/. Recommended formula 2 was used for the calculation. The average nucleotide identity (ANI) values were calculated using ANIcalculator v. 1.0 from enveomics collection (Rodriguez-R and Konstantinidis, 2016).

### Phylogenetic Analysis

The 16S rRNA sequences were aligned using MUSCLE included in MEGA 6.0 (Tamura et al., 2013). The maximum likelihood phylogenetic tree was computed by MEGA 6.0, using the Tamura-Nei substitution model and uniform rates among sites. Bootstrap tests were performed with 100 resamplings.

### 16S rRNA-Based Analysis of Microbial Community Composition

Raw reads obtained on GS FLX were screened against the RDP 16S rRNA database v. 11 (Cole et al., 2014) using BLASTN (*e*-value < 1e-5). Selected reads were passed to ssu_finder command of CheckM v. 1.05 to identify and extract the 16S rRNA gene sequences. A total of 881 identified sequences of 16S rRNA gene fragments were taxonomically assigned using the online RDP Naive Bayesian rRNA Classifier Version 2.0 with a confidence threshold of 0.8 (Wang et al., 2007).

### Nucleotide Sequence Accession Number

Metagenomic read data were deposited in the Sequence Read Archive (SRA) under the accession number SRR8953774. The annotated genome sequence of “*Candidatus* Jettenia ecosi J2” has been deposited in the GenBank database under accession number SULG00000000.

## Results

### Biomass Enrichment in a Lab-Scale Upflow Bioreactor

**FIGURE 1:**
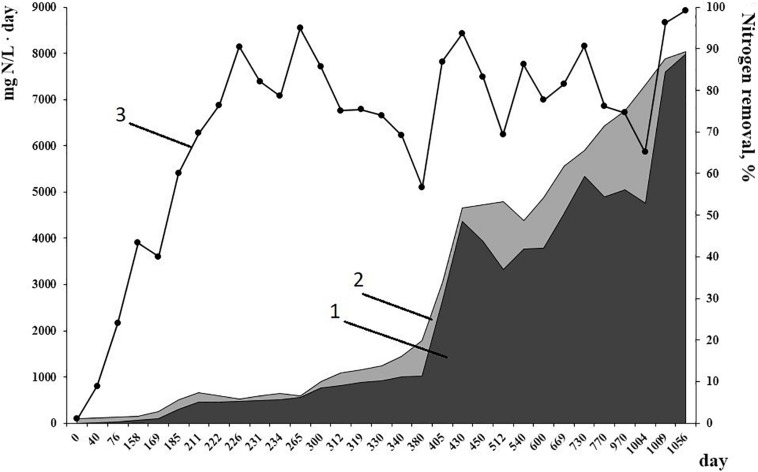
Nitrogen load (1) and nitrogen removal (2) rates, mg N/L⋅day, from 1 to 1056 day of reactor performance. (3) Efficiency of nitrogen removal, %.

Working conditions of the bioreactor (absence of active aeration and organics, high amounts of nitrogen substrates for anammox process) were selected to favor the growth of anammox bacteria and suppress the growth of other microbial groups. This resulted in the accumulation of a high amount of active biomass performing nitrogen removal. By the time metagenomic studies started, mean effectiveness of the anammox process had reached 96% (Figure 1). Inside the bioreactor, biofilms of various types were formed (Botchkova et al., 2014). Red-colored granules with a rigid surface, up to 7 mm in diameter were formed on the carriers and in the sediment. Thin (0.5 mm or less) easily broken biofilms developed on the walls of the bioreactor, on the surface of the effluent collector and inside the tubes for effluent removal. Also, single flocs occurred in the water column.

### FISH Analysis of the Microbial Community

**FIGURE 2:**
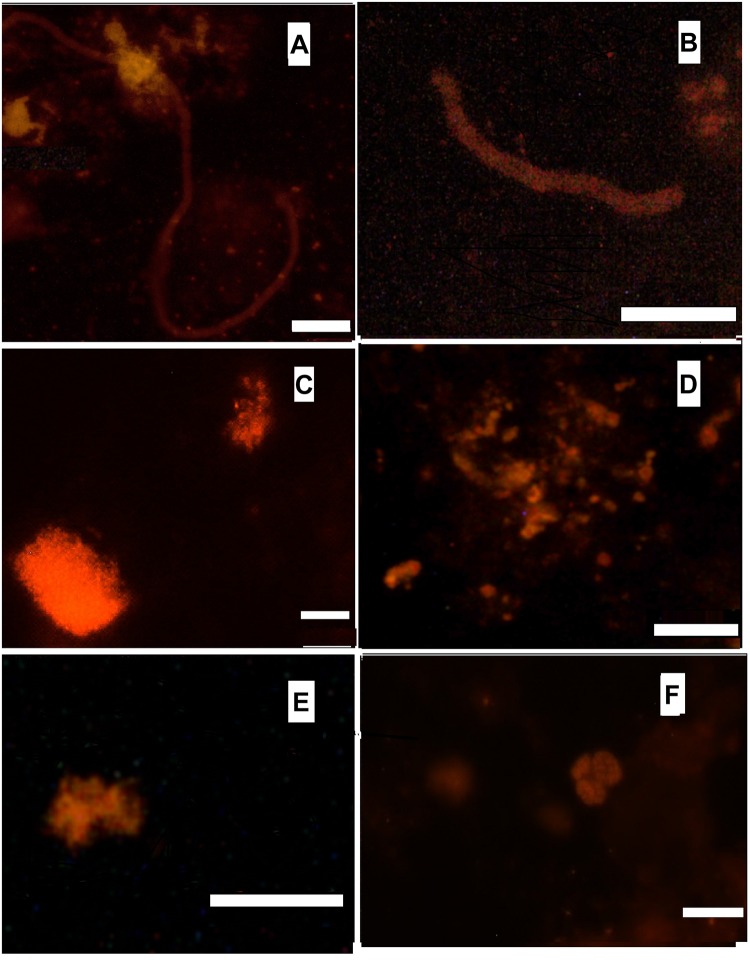
Hybridization with Cy-3 labeled probes. **(A)** CFX1223 (Phylum Chloroflexi), bar 10 μm; **(B)** CFX1223 (Phylum Chloroflexi), bar 5 μm; **(C)** NSE1472 (*Nitrosomonas* sp.), bar 5 μm; **(D)** EURY499 (methanogenic archaea), bar 10 μm; **(E)** Ntspn693 (*Nitrospina gracilis)*, bar 5 μm; **(F)** Ntspa662 (*Nitrospira sp.*), bar 10 μm.

FISH was used to visualize various groups of microbial satellites of anammox bacteria in the microbial community of the bioreactor, in order to complete and clarify the structure of the community. According to the results of phase contrast and electron microscopy, filamentous microorganisms are one of the most abundant members of the community (Botchkova et al., 2014). Usually they are observed outside the clusters of anammox cells, embedded into an extracellular polymeric matrix of the granules. Three distinct morphotypes of the filamentous microorganisms can be described: filaments 0.5 μm wide and up to 70 μm long; filaments 1–1.5 μm wide and up to 30 μm long; bead-shaped filaments 1 μm wide and up to 80 μm long. Filamentous microorganisms of the aforementioned two last morphotypes hybridized with probes with specificity to phylum Chloroflexi and thus can be attributed as members of this phylum (Figures 2A,B). Conditions inside the bioreactor are rather unfavorable for nitrifying microorganisms. Lack of active aeration was expected to suppress the growth of nitrifiers. However, both ammonium- and nitirite-oxidizing nitrifiers are present in the bioreactor community in a minor capacity, accounting for less than 5% of the total microbial population, according to the results of a direct count of hybridized cells. Aerobic ammonia oxidizers (AOB) of the genus *Nitrosomonas* were detected both in the granules and the flocs. They form densely packed clusters of approximately 30-40 coccoid cells (Figure 2C). Aerobic nitrite oxidizers (NOB) of the genera *Nitrospira* and *Nitrospina*, were also detected. They usually appear as small clusters or islets of 10–15 cells (Figures 2E,F). Application of the *Methanomicrobia-*specific probe EURY499 helped to trace the presence of methanogenic archaea. They form small single microcolonies in the core region of the granules (Figure 2D).

### Metagenome Assembly and Binning Results

In order to assemble the composite genomes of the most numerous members of the anammox community, we sequenced the metagenome of the red-colored anammox granules. A total of 682.6 Mb of metagenomic sequences were assembled into contigs, which were distributed among 13 major genome bins (metagenome-assembled genomes, MAGs), altogether comprised 77.4% of the whole metagenome (Table 2). Phylogenetic assignment of MAGs based on the searches against GTDB (Parks et al., 2018) revealed the members of five bacterial phyla, – *Planctomycetes* (43% of the whole metagenome), *Ignavibacteriae* (13%), *Chloroflexi* (9.9%), *Proteobacteria* (class beta, 9.2%), and *Armatimonadetes* (2.3%).

**TABLE 2:** General characteristics of MAGs obtained in this study.

**Bin Id**	**Phylogenetic assignment**	**Completeness (%)**	**Redundancy (%)**	**Contigs**	**Genome size (Mbp)^∗^**	**Genome coverage**	**Share in the whole metagenome (%)**
2	*Planctomycetes; Ca*. Jettenia	100.0	3.5	223^∗∗^	3.86^∗∗^	53	30.53
15	*Planctomycetes; Ca*. Brocadia	90.1	9.3	867	3.55	8.0	4.15
17	*Planctomycetes; Ca*. Kuenenia	25.6	0.1	1298	2.03	7.0	2.08
16	*Planctomycetes; Phycisphaerales*	67.2	6.5	1404	3.51	6.0	3.10
20	*Planctomycetes; Phycisphaerales*	80.5	1.8	603	3.27	6.4	3.09
1	*Ignavibacteriae; Ignavibacterium*	86.0	0	73	3.18	27	12.46
21	*Ignavibacteriae; Ignavibacterium*	30.7	0	649	1.05	3.5	0.54
11	*Chloroflexi; Anaerolineae; Ca*. Promineofilaceae	95.3	4.7	436	5.82	9.7	8.22
3	*Chloroflexi; Anaerolineae*	53.1	2.0	1222	2.80	4.2	1.72
10	*Betaproteobacteria; Burkholderiaceae*	85.9	1.3	542	3.21	13	5.97
4	*Betaproteobacteria; Burkholderiaceae*	25.6	3.1	1057	1.49	5.9	1.28
7	*Betaproteobacteria; Rhodocyclaceae*	68.3	29.7	2152	3.74	3.6	2.00
9	*Armatimonadetes; Fimbriimonadaceae*	73.4	3.3	924	2.95	5.2	2.26

*^∗^Total length of all contigs; ^∗∗^upon manual curation of this bin.*

**FIGURE 3:**
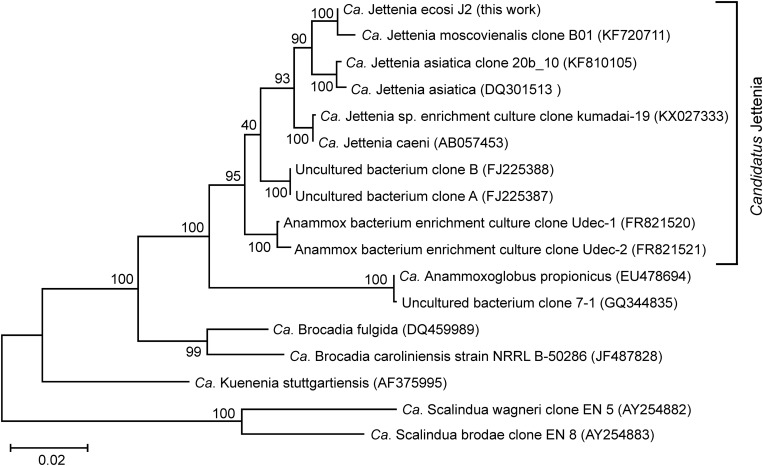
Position of “*Candidatus* Jettenia ecosi” J2 on maximum likelihood 16S rRNA gene phylogenetic tree. GenBank accession numbers are shown after the clone names. The scale bar represents substitutions per nucleotide base. Bootstrap values are indicated at the nodes.

The most abundant phylum, *Planctomycetes*, was represented by five MAGs. Three of them were assigned to anammox genera “*Ca.* Jettenia,” “*Ca.* Brocadia” and “*Ca.* Kuenenia,” while two other MAGs belonged to the order *Phycisphaerales* but were phylogenetically distant from known genera. A single MAG, designated J2 and assigned to the genus “*Ca.* Jettenia,” accounted for 30.5% of the whole metagenome. It was sequenced to 53x average coverage and represented by 223 contigs with a total length of 3,864,554 bp. CheckM estimated the completeness of this genome as 100%, with 3.4% possible contamination (redundancy). Therefore this MAG met the recently proposed criteria (Bowers et al., 2017) for the high quality metagenome-assembled genomes (> 90% completeness with < 5% redundancy and the presence of rRNA genes). The 16S rRNA sequence present in this MAG was 100% identical to that of “*Candidatus* Jettenia ecosi,” an anammox bacterium previously found in the studied bioreactor (Botchkova et al., 2018). It also shared a 98.3% sequence identity with “*Candidatus* Jettenia asiatica” AS-1 (Quan et al., 2008) and 98.3% with “*Candidatus* Jettenia caeni” KSU-1 (Ali et al., 2015). A 99.4% sequence identity was found for 16S rRNA gene sequences of the J2 bacterium and “*Candidatus* Jettenia moscovienalis” clone B01 (Nikolaev et al., 2015), although only a 644-bp long 16S rRNA gene fragment was reported for this tentative species (GenBank KF720711). Phylogenetic analysis based on 16S rRNA gene sequences placed J2 bacterium within the genus “*Candidatus* Jettenia,” where it formed a distinct lineage along with candidate species “Jettenia caeni” and “Jettenia asiatica” (Figure 3). Among the members of the genus “*Candidatus* Jettenia,” genome sequence is available only for “*Candidatus* Jettenia caeni” KSU-1 (GenBank NZ_BAFH00000000); the value of *in silico* DNA-DNA hybridization of J2 and KSU-1 genomes was about 60%, which does not allow whether these bacteria belong to a single or different species to be defined. The ANI between the genomes of J2 and KSU-1 was 94.91%, a value just below the species boundary cutoff of 95% (Konstantinidis and Tiedje, 2005; Jain et al., 2018), suggesting a recent divergence and speciation of these species (Olm et al., 2019).

The second most abundant phylum, *Ignavibacteriae*, was represented by MAG 1 with an estimated completeness of 86% and no detected redundancy, and a poorly assembled MAG 21 representing less abundant organism (Table 2). MAG1, designated Ignavibacteriales bacterium MGE-1, was phylogenetically close to Ignavibacteriales bacterium UTCHB2 (84.9% AAI), identified in an anammox bioreactor (Lawson et al., 2017), and more distantly related to two cultured species of this phylum, *Ignavibacterium album* (64.4% AAI) and *Melioribacter roseus* (53.8% AAI). Both these species are facultative anaerobes growing on carbohydrates by aerobic respiration, fermentation or by reducing diverse electron acceptors (Liu et al., 2012; Kadnikov et al., 2013). According to the AAI thresholds for classification of uncultivated microorganisms (45–65% for the same family, 65–95% for the same genus and 95–100% for the same species, Konstantinidis et al., 2017), Ignavibacteriales bacterium MGE-1 and UTCHB2 belongs to the same genus within the family Ignavibacteriaceae. Genome analysis of Ignavibacteriales bacterium MGE-1 revealed that this is metabolically versatile heterotrophic microorganism with capacities for fermentation, aerobic and anaerobic respiration. Particularly, the genome contained *narGHIJ* genes coding for membrane-linked respiratory nitrate reductase that reduce nitrate to nitrite and genes for pentaheme nitrite reductase NrfHA reducing nitrate to ammonia. In addition, nitrous oxide reductase could perform dissimilatory reduction of N_2_O to dinitrogen gas, while genes for the nitric oxide reductase (*norBC* or *norZ*) were not found. Terminal oxygen reductases in MGE-1 bacterium are represented by a low-affinity *b(o/a)3*-type cytochrome c oxidase and a high-affinity quinol oxidase *bd* complex.

The phylum *Chloroflexi* was represented by two MAGs. More abundant MAG11, assembled with an estimated 95.5% completeness and 4.7% redundancy, was phylogenetically related to *Ca.* Promineofilum breve (56.2% AAI), detected in activated sludge of wastewater treatment plant (McIlroy et al., 2016). Genome of this bacterium, designated Chloroflexi bacterium MGE-11, encoded NarGHIJ respiratory nitrate reductase, NrfHA nitrite reductase and nitrous oxide reductase. Contrary to Ignavibacteriales bacterium MGE-1, the MGE-11 genome also contained the *nirK* gene of copper-containing nitrite reductase that reduces nitrite to nitric oxide. Complete aerobic respiratory chain is also present, including NADH dehydrogenase, succinate dehydrogenase, cytochrome *bc* complex III and cytochrome *c* oxidase, as well as a quinol oxidase *bd* complex.

Three MAGs were assigned to the families *Burkholderiaceae* and *Rhodocyclaceae* of the Betaproteobacteria, but appeared to be phylogenetically distant from cultured species. In addition, a single MAG was assigned to the family *Fimbriimonadaceae* of the phylum *Armatimonadetes* (Table 2).

### Anammox Metabolic Pathways of “*Candidatus* Jettenia ecosi J2”

Genome analysis of “*Ca*. Jettenia ecosi” J2 revealed pathways rather typical for anammox bacteria (reviewed in Kartal and Keltjens, 2016). Anammox bacteria should be able to take up substrates, ammonium and nitrite, used for the anammox process from the environment. Consistently, the genome of “*Ca*. Jettenia ecosi” J2 encodes nine copies of AmtB-like ammonium transporters. Four of these genes are clustered with genes-encoding nitrogen regulatory proteins P-II that bind directly to the AmtB and regulate the ammonia channel (Conroy et al., 2007). Three *focA* genes encoding nitrite/formate transporters were identified, along with a single gene for a putative nitrate/nitrite transporter NarK.

The first step of the anammox reactions, reduction of nitrite to NO, was thought to be catalyzed by a cytochrome *cd*_1_-type nitrite reductase NirS in the “*Candidatus* Kuenenia*”* and “*Candidatus* Scalindua*”* species (Strous et al., 2006; van de Vossenberg et al., 2013), while the copper-containing NirK nitrite reductase was found in the genomes of the anammox strain KSU-1, “*Candidatus* Jettenia asiatica” and “*Candidatus* Brocadia fulgida” (Hira et al., 2012; Hu et al., 2012). Gene encoding NirK-type nitrite reductase was identified in the genome of “*Ca*. Jettenia ecosi” J2.

The nitric oxide, produced from nitrite by nitrite reductase NirK, and ammonium taken from the environment, are used by a hydrazine synthase to produce hydrazine (N_2_H_4_) (Kartal et al., 2011). The three-gene operon coding for HszABC hydrazine synthase, was found in “*Ca*. Jettenia ecosi” J2. The final step of the anammox process, the oxidation of hydrazine with the production of gaseous N_2_, is catalyzed by octahaem hydrazine dehydrogenase (HDH) (Kartal et al., 2011).

Interconversion of nitrate and nitrite could be enabled by a nitrate reductase. The genome of “*Ca*. Jettenia ecosi” J2 contained genes for subunits alpha, beta and gamma of NarGHI-type nitrate reductase and nearly located gene encoding chaperone protein TorD. Although nitrate reductase typically reduces nitrate to nitrite, it was proposed that in anammox bacteria it runs in the reverse direction and performs the oxidation of nitrite to nitrate, providing the reducing equivalents for CO_2_ fixation (Jetten et al., 2009). However, recent studies of *Ca.* K. stuttgartiensis culture grown in bioreactor supplied with ammonium and NO as the only substrates suggested that the low potential electrons released from hydrazine oxidation to N_2_ are directed to cell carbon fixation, while nitrite oxidation to nitrate and nitrite reduction to NO are probably coupled to one another (Hu et al., 2019).

**FIGURE 4:**
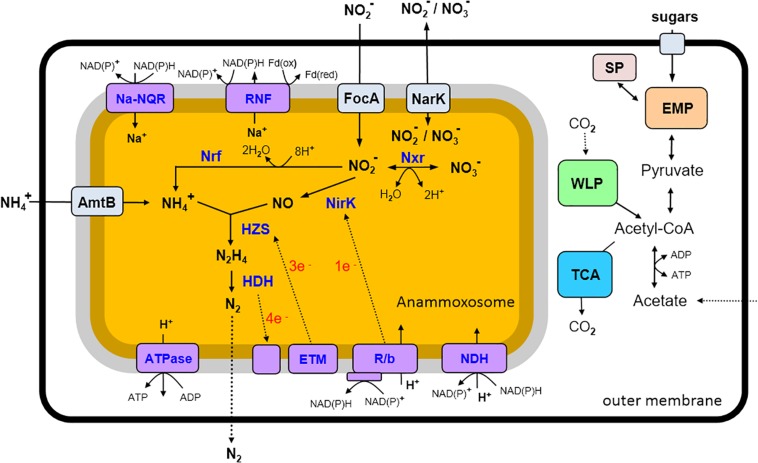
An overview of predicted metabolic pathways of “*Candidatus* Jettenia ecosi” J2. Nxr, nitrite:nitrate oxidoreductase; Nrf, nitrite reductase forming ammonium; NirK, nitrite reductase; HZS, hydrazine synthase; HDH, hydrazine dehydrogenase; ETM, electron transfer module from the quinone pool to HZS; R/b, Rieske/cytochrome *b* (*bc1*) complexes; NDH, NADH dehydrogenase; ATPase, ATP synthase; RNF, RNF complex; Na-Nqr, Na-translocating NADH-quinone oxidoreductase; AmtB, ammonium transporters; FokA, nitrite transporters; NarK, nitrite/nitrate transporter; SP, storage polysaccharides; EMP, Embden–Meyerhof–Parnas pathway; WLP, Wood–Ljungdahl pathway; TCA, tricarboxylic acid cycle.

The presence of nitrate reductase suggests that “*Ca*. Jettenia ecosi” J2 could reduce nitrate to nitrite followed by the dissimilatory reduction of nitrite to ammonium by the NrfAH cytochrome *c* nitrite reductase encoded in the genome, as it was shown for purified *Ca.* K. stuttgartiensis cells (Kartal et al., 2007). The products of this pathway, nitrite and ammonium, may in turn drive the anammox process. An overview of predicted metabolic pathways of “*Ca*. Jettenia ecosi” J2 is shown in Figure 4.

### Other Important Metabolic Pathways of the J2 Bacterium

Anammox bacteria are able to grow autotrophically and perform carbon fixation via the Wood–Ljungdahl pathway (Schouten et al., 2004). Consistently, a complete set of genes encoding the enzymes of this pathway, is present in “*Ca*. Jettenia ecosi” J2 genome, namely formate dehydrogenase, formate-tetrahydrofolate ligase, methylenetetrahydrofolate dehydrogenase/methenyltetrahydrofolate cyclohydrolase, 5,10-methylenetetrahydrofolate reductase, 5-methyltetrahydrofolate:- corrinoid iron–sulfur protein methyltransferase, and the carbon monoxide dehydrogenase/acetyl-CoA synthase complex.

The major electron transport chain components were identified in the “*Ca*. Jettenia ecosi” J2 genome, namely translocating NADH-dehydrogenase complex, Rieske-heme *b* complexes (R/b; *bc*_1_ complexes), and an F_0_F_1_-type H^+^-transporting ATPase. The transmembrane ion gradient could be also generated by a Na^+^-translocating NADH-quinone oxidoreductase, Na-NQR, identified as the main ion pump in different pathogenic and free-living bacteria (Reyes-Prieto et al., 2014). An operon coding for all six subunits of this complex (NqrA-F, with fused D and E subunits) was found in the genome. The genome also contained a genes encoding membrane-bound ion-transporting complex Rnf, evolutionary related to Na-NQR. This complex could act as an energy-conserving ferredoxin: NAD^+^ oxidoreductase coupling oxidation of ferredoxin to NAD^+^ reduction and translocation of Na^+^ ions or protons or sodium ions across the membrane (Biegel et al., 2011). The presence of above mentioned ion-translocating complexes in *Ca.* K. stuttgartiensis cell was recently detected by proteomics-based complexome profiling (de Almeida et al., 2016).

The presence of a complete Embden-Meyerhof pathway (EMP) of glycolysis and downstream enzymes, pyruvate-flavodoxin oxidoreductase, and acetate kinase, suggested that J2 bacterium could be capable of fermentation of carbohydrates with the concomitant generation of ATP. Acetyl-CoA could be also produced from acetate by ADP-forming acetyl-CoA synthetase. In course of autotrophic growth the EMP operates in the direction of gluconeogenesis, consistently with the presence of phosphoenolpyruvate synthase and fructose-1,6-bisphosphatase. This pathway is probably also involved in the synthesis and degradation of storage polysaccharides, trehalose and glycogen. Enzymes of two pathways of trehalose synthesis were encoded: the trehalose synthase TreS, and two enzymes of the TreYZ pathway, malto-oligosyltrehalose synthase TreY and trehalohydrolase TreZ. A set of enzymes required for glycogen synthesis and hydrolysis is encoded as well, including glycogen synthase, branching and debranching enzymes, and glycogen phosphorylases. Genome analysis revealed no carbohydrate-active enzymes that carry N-terminal signal peptides indicating their involvement in the extracellular hydrolysis of polysaccharides. However, the presence of a maltose/maltodextrin ABC transporter suggests the possibility that these carbohydrates could be imported from the environment and support heterotrophic growth.

Genome analysis revealed two [NiFe] hydrogenases indicating that hydrogen turnover could play important role in metabolism. The first belongs to group 3b of cytosolic bidirectional hydrogenases that couples oxidation of NADPH to evolution of H_2_ (Greening et al., 2016). The second enzyme is a multisubunit membrane-linked group 4f respiratory H_2_-evolving hydrogenase. Such enzymes can form respiratory complex that couples oxidation of formate to proton reduction and translocate protons across the membrane through the antiporter-like subunits thus conserving energy in the form of proton gradient (Greening et al., 2016). Formate could be generated in the Wood–Ljungdahl pathway in course of the autotrophic growth or acquired from the environment.

### Genome Comparison of “*Ca*. Jettenia ecosi” and “*Ca*. Jettenia caeni”

Pairwise genome comparisons of “*Ca*. Jettenia ecosi” and “*Ca*. Jettenia caeni” revealed that 2796 of 3914 protein-coding genes of “*Ca*. Jettenia ecosi” are also present in “*Ca*. Jettenia caeni.” Among 1118 genes specific to “*Ca*. Jettenia ecosi” most were annotated as encoding hypothetical proteins (956), components of restriction-modification systems (20) and CRISPR-associated proteins (10). A notable difference between two genomes that could impact the metabolic properties is the absence in “*Ca*. Jettenia caeni” of approximately 40 kb long fragment present in “*Ca*. Jettenia ecosi” (genes JETT_0044-JETT_0074). This region comprised several genes important for the glycolysis and downstream pathways, including class II fructose-bisphosphate aldolase, glucose-6-phosphate isomerase and acetate kinase. Homologs of these genes are present in the genomes of *Ca.* Brocadia sp. and other *Planctomycetes*. Since the flanking regions are collinear in the genomes of “*Ca*. Jettenia ecosi” and “*Ca*. Jettenia caeni,” this gene cluster has been probably lost in “*Ca*. Jettenia caeni.”

### Microbial Community Composition Revealed by 16S rRNA Data

Numerous studies of anammox bioreactors revealed complex microbial communities comprising, in addition to dominant anammox planctomyctes, a number of such physiological groups, as fermentative bacteria, nitrifiers, denitrifiers, methanogens, and others (Hu et al., 2012; Gonzalez-Martinez et al., 2015a; Speth et al., 2016; Mardanov et al., 2017; Park et al., 2017; Zhao Y. et al., 2018). In order to get deeper insights into microbial community composition and reveal minor community members for which MAGs were not assembled, we analyzed raw pyrosequencing reads. Mapping of the raw reads to the RDP database revealed 881 reads representing the 16S rRNA gene fragments, of which 798 were taxonomically assigned. *Planctomycetes* accounted for 61.2% of the assigned reads and were mostly represented by the family “*Candidatus* Brocadiaceae” (55.8%). The search for 16S rRNA sequences in metagenomic contigs revealed, in addition to the dominant “*Ca*. Jettenia ecosi” J2, two other anammox planctomycetes present in minor amounts – “*Candidatus* Kuenenia stuttgartiensis” (100% identity of 16S rRNA gene sequences) and “*Candidatus* Brocadia sp. 40” (97% identity). Two other 16S rRNA sequences of *Planctomycetes* found in the contigs were assigned to the non-anammox order *Phycisphaerales*. The same lineages were identified as a result of metagenome binning.

The second most abundant group was the phylum *Ignavibacteriae* (14.2% of the assigned reads). *Proteobacteria* accounted for 15.2% of the assigned reads and mostly belonged to classes beta (11.2%) and gamma (3.2%). Four beta-proteobacterial 16S rRNA sequences found in the contigs were assigned to the genera *Comamonas, Zhizhongheella, Dechlorobacter*, and *Denitratisoma*. The presence of the latter two species suggests that the processes of dissimilatory reduction of perchlorate compounds and nitrate could play an important role in the anammox reactor. Gamma-proteobacteria belong to the genera *Pseudomonas* and *Thermomonas*, metabolically versatile heterotrophic bacteria. Other abundant community members belong to the *Chloroflexi* of the class *Anaerolinea* (4.3% of reads), the phylum *Armatimonadetes* (2.1%), and *Firmicutes* (1.3%). Methanogenic archaea, often present in anammox bioreactors (Hu et al., 2012), were not found.

## Discussion

The structure of the microbial community of lab-scale bioreactors based on the anammox process is usually rather complex and includes numerous phylotypes of microorganisms. They are connected with each other by complex and, in most cases, poorly understood relations. However, recent metagenomic and metatranscriptomic studies reveal the existence of a core microbiome in the microbial communities of anammox-based bioreactors, which is shown to be similar in various types of reactor despite differences in reactor operation and influent wastewater composition (Gonzalez-Martinez et al., 2015b; Speth et al., 2016; Lawson et al., 2017). According to metatranscriptomic studies, most of the heterotrophic members of the community are involved in N-cycle processes and can carry out partial denitrification (Speth et al., 2016; Lawson et al., 2017).

Metagenomic analysis of anammox granules revealed complex microbial community dominated by anammox *Planctomycetes* and also containing members of *Ignavibacteriae*, *Chloroflexi*, *Proteobacteria*, and *Armatimonadetes* and non-anammox *Planctomycetes*. Although usually either *Ca*. Brocadia or *Ca*. Kuenenia dominated in lab-scale anammox bioreactors (Hu et al., 2010; Park et al., 2010) and often out-competed *Ca*. Jettenia (Zhao et al., 2019), we detected the presence of all three anammox species and the dominance of “*Ca*. Jettenia ecosi” J2. It is possible that relatively stable operation conditions and high nitrogen load are more favorable for *Ca*. Jettenia since Ca. Brocadia was shown to out-compete it at low nitrogen concentrations (Zhao et al., 2019).

Genome analysis of “*Ca*. Jettenia ecosi” J2 revealed metabolic pathways, related to anammox reactions and autotrophic carbon fixation, previously reported for “*Ca.* Jettenia asiatica” (Hu et al., 2012) and other anammox *Planctomycetes* (reviewed in Kartal et al., 2013 and Kartal and Keltjens, 2016). In addition, this analysis suggested that “*Ca*. Jettenia ecosi” could grow heterotrophically. The presence of acetyl-CoA synthetase and nitrate/nitrite reductases indicated the possibility of respiratory ammonification with oxidation of acetate, as observed for “*Ca*. Jettenia caeni” (Ali et al., 2015). Genome encodes all the enzymes required for the synthesis and degradation of storage polysaccharides that could be used for fermentative metabolism in the absence of substrates for anammox process. Moreover, the finding of sugar ABC-type transporters suggests that “*Ca*. Jettenia ecosi” could obtain sugars from the environment. Acetate and hydrogen could be produced in course of fermentative growth.

The second most numerous bacterial lineage, the phylum Ingavibacteriae, is omnipresent in anammox bioreactors (Li et al., 2009; Park et al., 2010; Gonzalez-Gil et al., 2015; Gonzalez-Martinez et al., 2015a, b; Speth et al., 2016) and were considered as protein degraders, catabolizing extracellular peptides while reducing nitrate to nitrite (Lawson et al., 2017). The dominant member of this phylum, MGE-1, was phylogenetically close to Ignavibacteriales bacterium UTCHB2, identified in an anammox bioreactor by Lawson et al. (2017). Genome analysis of MGE-1 bacterium suggested that it can grow heterotrophically, reducing nitrate to nitrite and then nitrite to ammonia thus providing substrates for anammox bacteria. Filamentous bacteria of the phylum *Chloroflexi* usually account for a significant part of the microbial community of anammox-based bioreactors (Kindaichi et al., 2012; Chu et al., 2015; Wang et al., 2017) and were also detected in this study both by metagenomics and FISH analysis (Figures 2A,B). It is hypothesized that in anammox bioreactor communities *Chloroflexi* are involved in the degradation of organics from dead cells, or they can be involved in the synthesis or degradation of extracellular polymeric matrix (Ni et al., 2012; Speth et al., 2016; Zhao Y. et al., 2018). Due to their filamentous structure, they play an important role in the formation of biofilms (Kindaichi et al., 2012; Zhao Y. et al., 2018). Genome analysis of Chloroflexi bacterium MGE-11 revealed that, like Ignavibacteriales bacterium MGE-1, this species could grow heterotrophically and perform dissimilatory reduction of nitrate and nitrite. In addition MGE-11 has the capacities to reduce nitrite to NO, therefore performing the first step of an anammox process. Interestingly, both MGE-1 and MGE-11 genomes encoded the nitrous oxide reductase responsible for reduction of N_2_O to N_2_. Nitrous oxide could be produced from NO by the nitric oxide reductase. The corresponding genes were not found in these genomes but could be present in other community members, for example, Proteobacteria and Bacteroidetes (Lawson et al., 2017).

The results of FISH complemented the metagenomic studies since metagenomics cannot substitute for the information that can be gained by visualizing the identity and activity of single microbial cell *in situ*. Moreover, in frame of the present study metagenomics was unable to reveal minor members of the community due to limited sequencing volume. In some cases, even populations of a relative abundance of 1 in 1,000 cells can be accurately quantified (Amann and Fuchs, 2008). Several important microbial minor groups were detected and visualized only by FISH, – members of the genera *Nitrosomonas* (beta-proteobacteria), *Nitrospina* (phylum *Nitrospinae*), *Nitrospira* (phylum *Nitrospirae*), and methanogenic archaea.

Members of the genus *Nitrosomonas* were reported to coexist with anammox bacteria in bioreactor communities, the numbers of which were shown to decrease with the growth in the size of granules (Dosta et al., 2015; Gonzalez-Martinez et al., 2015b; Liu et al., 2017). Clusters of cells, hybridized with probe NSE1472, are about 7-10 μm in diameter, densely packed and morphologically resemble clusters of AOB cells discovered in MBBR with partial nitritation-anammox (Almstrand et al., 2014; Persson et al., 2017).

NOB were rarely detected in anammox bioreactors with microaerophilic or anaerobic conditions since they are more sensitive than AOB to lack of aeration (Dosta et al., 2015; Speth et al., 2016). However, in some cases NOB occur in anammox bioreactors even under anaerobic conditions (Liu et al., 2017), mainly members of the genus *Nitrospira* which were shown to carry out the commamox process (van Kessel et al., 2015; Mardanov et al., 2016).

Obligate anaerobes, methanogenic archaea of the class *Methanomicrobia*, were also present in the community. According to previous studies, methanogenes exist even in bioreactors operated under microaerophilic conditions or in two-stage reactors with alternating aerobic-anaerobic conditions (Gonzalez-Gil et al., 2015; Pereira et al., 2017). In our case, the core parts of the granules provide suitable conditions (the absence of oxygen) for members of *Methanomicrobia* to exist.

## Data Availability Statement

Metagenomic read data were deposited in the Sequence Read Archive (SRA) under the accession number SRR8953774. The annotated genome sequence of “*Candidatus* Jettenia ecosi J2” has been deposited in the GenBank database under accession number SULG00000000 (https://www.ncbi.nlm.nih.gov/nuccore/SULG00000000).

## Author Contributions

AM and AB performed methagenomic studies – methagenome sequencing and assembly, contig binning, and analysis of the composite genome of the anammox bacterium. EB and YL performed anammox bioreactor operation and conducted chemical analyses, bacteria sampling, and FISH analysis and microscopy. AN and NR edited the final draft of the manuscript and were responsible for the general direction of the work and development of general strategies. All authors equally participated in the manuscript writing and discussion.

## Conflict of Interest

The authors declare that the research was conducted in the absence of any commercial or financial relationships that could be construed as a potential conflict of interest.

## Footnotes

1 http://www.cbs.dtu.dk/services/SignalP/

2 http://www.compgen.org/tools/PRED-TAT/

3 http://www.cbs.dtu.dk/services/TMHMM/

## Supplementary Material

FIGURE S1 Granules from the bioreactor. Bar 10 mm.

TABLE S1 List of oligonucleotide probes used in the current study, and their specificity (according to http://probebase.csb.univie.ac.at/).
