# Supplementary figures and images for: Genome of a Novel Bacterium “Candidatus Jettenia ecosi” Reconstructed From the Metagenome of an Anammox Bioreactor
Source: Front Microbiol. 2019 Oct 29;10:2442. doi: 10.3389/fmicb.2019.02442 (PMC6828613; doi:10.3389/fmicb.2019.02442)

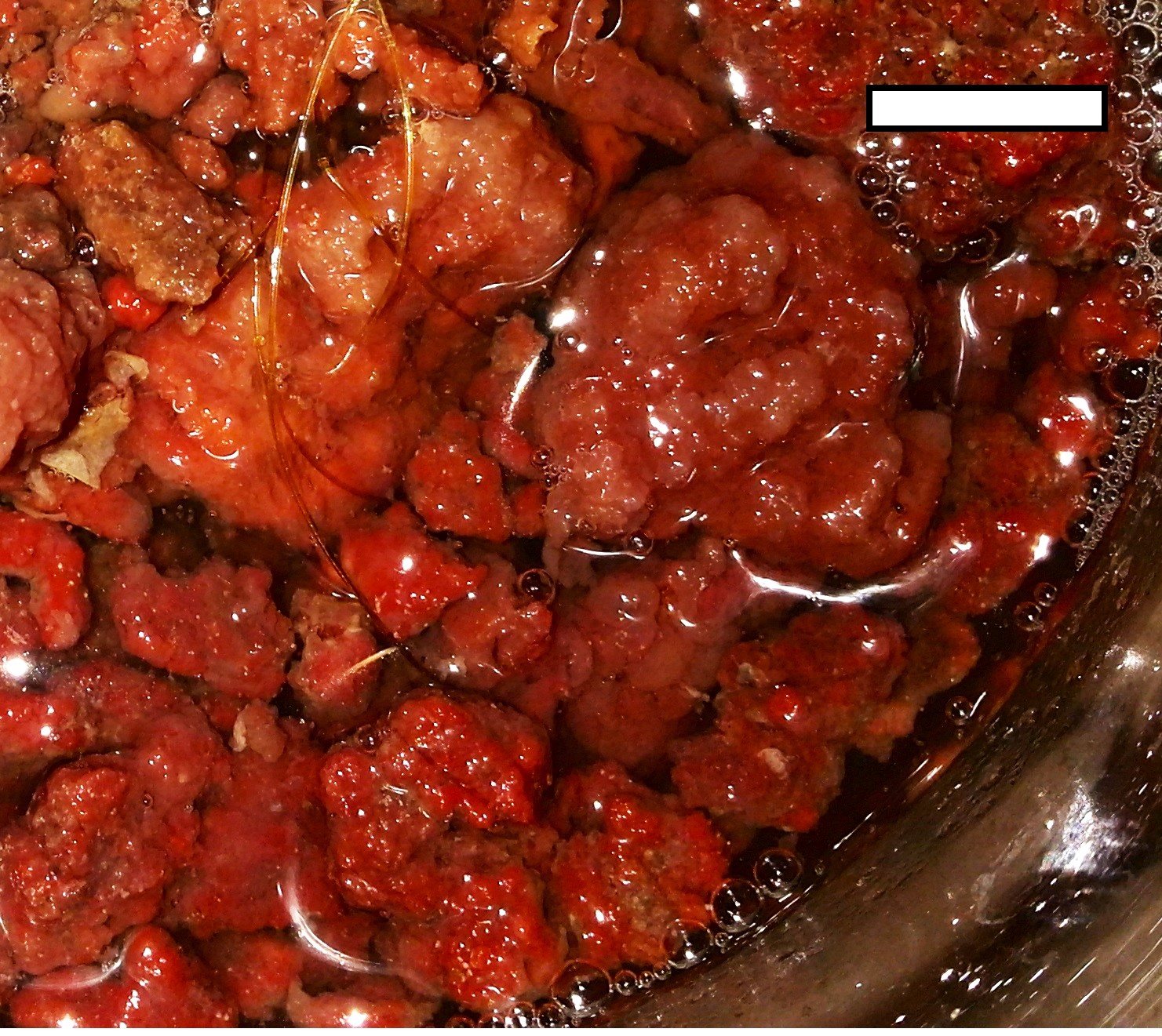

Supplement: FIGURE S1 — Granules from the bioreactor. Bar 10 mm. [file Image_1.JPEG]
