# Supplementary material for: Genome of a Novel Bacterium “Candidatus Jettenia ecosi” Reconstructed From the Metagenome of an Anammox Bioreactor
Source: Front Microbiol. 2019 Oct 29;10:2442. doi: 10.3389/fmicb.2019.02442 (PMC6828613; doi:10.3389/fmicb.2019.02442)
Supplement: TABLE S1 — List of oligonucleotide probes used in the current study, and their specificity (according to http://probebase.csb.univie.ac.at/). [file Table_1.DOCX]

**Supplementary materials**

Table S1. List of oligonucleotide probes used in the current study, and their specificity (according to <http://probebase.csb.univie.ac.at/>)

| **Name of the probe** | **Target microbial group** | **[FA], %** | **Nucleotide sequence** |
| --- | --- | --- | --- |
| CFX1223 | Phylum Chloroflexi | 35 | **5'-**CCA TTG TAG CGT GTG TGT MG **-3'** |
| NSE1472 | *Nitrosomonas europea,*  *N. halophila,*  *N. eutropha* | 50 | **5'-**ACC CCA GTC ATG ACC CCC **-3'** |
| Ntspn693 | *Nitrospina gracilis* | 20 | **5'-**TTC CCA ATA TCA ACG CAT TT **-3'** |
| Ntspa662 | Genus *Nitrospira* | 35 | 5'-GGA ATT CCG CTC TCC TCT -3' |
| EURY499 | *Methanosarcina, Methanosaeta, Methanomicrobiales* and clonesVAL 47,VAL 1, VAL9, VAL78 | 20 | **5'-**CGG TCT TGC CCG GCC CT **-3'** |
